# *MYCN* Amplifications and Metabolic Rewiring in Neuroblastoma

**DOI:** 10.3390/cancers15194803

**Published:** 2023-09-29

**Authors:** Marialena Pouliou, Marianna A. Koutsi, Lydia Champezou, Angeliki-Ioanna Giannopoulou, Giannis Vatsellas, Christina Piperi, Marios Agelopoulos

**Affiliations:** 1 Center of Basic Research, Biomedical Research Foundation, Academy of Athens, 4 Soranou Ephessiou St., 11527 Athens, Greece; mpouliou@bioacademy.gr (M.P.); mkoutsi@bioacademy.gr (M.A.K.); lchamp@bioacademy.gr (L.C.); gvatsellas@bioacademy.gr (G.V.); 2 Department of Biological Chemistry, Medical School, National and Kapodistrian University of Athens, 75 M. Asias Street Bldg 16, 11527 Athens, Greece; angelig@med.uoa.gr

**Keywords:** cancers, neuroblastoma, metabolism, dysregulated gene expression, transcription factors, *MYCN* amplifications, genomics, computational investigations

## Abstract

**Simple Summary:**

Transcription factors (TFs) can reprogram cellular states by modulating the expression of their target genes and establishing gene expression programs under homeostasis and diseases manifestation. In neuroblastoma, the TF MYCN has been recorded as dysregulated, presenting both aberrant expression and genomic abnormalities across its coding locus. Herein, we computationally investigated the gene expression characteristics that distinguish neuroblastoma-*MYCN*-amplified from neuroblastoma non-*MYCN*-amplified cancer cells, and we addressed the upregulation of several metabolism-related TF-encoding genes. Moreover, cistromic computational assessments of MYCN revealed its direct binding across regulatory sequences that reside in cis proximity to several of those genes. These results illuminate substantial mechanistic interrelationships between the key driver of neuroblastoma and a wealth of transcriptional regulators in cancer cells.

**Abstract:**

Cancer is a disease caused by (epi)genomic and gene expression abnormalities and characterized by metabolic phenotypes that are substantially different from the normal phenotypes of the tissues of origin. Metabolic reprogramming is one of the key features of tumors, including those established in the human nervous system. In this work, we emphasize a well-known cancerous genomic alteration: the amplification of *MYCN* and its downstream effects in neuroblastoma phenotype evolution. Herein, we extend our previous computational biology investigations by conducting an integrative workflow applied to published genomics datasets and comprehensively assess the impact of *MYCN* amplification in the upregulation of metabolism-related transcription factor (TF)-encoding genes in neuroblastoma cells. The results obtained first emphasized overexpressed TFs, and subsequently those committed in metabolic cellular processes, as validated by gene ontology analyses (GOs) and literature curation. Several genes encoding for those TFs were investigated at the mechanistic and regulatory levels by conducting further omics-based computational biology assessments applied on published ChIP-seq datasets retrieved from *MYCN*-amplified- and *MYCN*-enforced-overexpression within in vivo systems of study. Hence, we approached the mechanistic interrelationship between amplified *MYCN* and overexpression of metabolism-related TFs in neuroblastoma and showed that many are direct targets of MYCN in an amplification-inducible fashion. These results illuminate how *MYCN* executes its regulatory underpinnings on metabolic processes in neuroblastoma.

## 1. Introduction

Cancer is defined by genomic, epigenomic, and gene expression abnormalities [1] and is characterized by the evolution of metabolic phenotypes that are substantially deviated from those of the original tissues [2,3]. In cancerous states, essential cellular functions connected to bioenergetics, biosynthesis, redox balance, and other dependencies are modified compared to normal cells. These cellular decisions facilitate the increased nutrient uptake and tolerance within tumor microenvironments [3,4]. In many cases, diverse tumors share metabolic properties that are supported by common cellular pathways. These findings have led to a revolution in the cancer metabolism field, which then introduced metabolic reprogramming as a hallmark of tumorigenesis [3,5,6]. At the cellular level, metabolic rewiring underpins growth, survival, and proliferation of tumors [2,7]. Accordingly, metabolism-related gene expression signatures correlate with patient survival [8]. For instance, one of the key metabolic features of cancerous cellular states is excessive glucose uptake and its fermentation to lactate [7]. This functional shifting from oxidative phosphorylation to aerobic glycolysis is the renowned “Warburg effect” [7,9,10,11], which describes how cancer cells excessively produce lactate as a derivative of glucose, regardless of oxygen levels. This mechanistic adaptation is vital for long-term tumor sustenance since it enables cancer cells to fine-tune ATP synthesis [11]. It is noteworthy that the metabolic features of cancerous cellular states are not stable but rather change during tumor evolution, thus leading to “metabolically heterogeneous” phenotypes [2,12]. A wealth of examples describes the rewiring of pathways between later and initial stages of cancer phenotypes [13,14,15]. For example, aberrant activation of EGFR in non-small-cell lung carcinoma (NSCLC) drives UDP-glucose 6-dehydrogenase (UGDH) phosphorylation and activation. UGDH abolishes UDP-glucose resulting in enhanced SNAI1 mRNA stability. Increased production of SNAI1 transcription factor (TF) induces the epithelial-to-mesenchymal transition enabling cancer cell migration and metastasis [15]. Together, the above discoveries imply the constant function of cellular mechanisms of gene expression regulation that can confer adaptation to the “newly evolved” tumor microenvironments during the initiation, growth, progression, and metastasis of human cancers.

Tumors are addicted to transcriptional dysregulation [1,16] that is orchestrated by transcription factors (TFs) and featured as aberrant gene expression programs [1,17]. TFs are the endpoints where signal transduction pathways converge [18] and they interpret DNA grammar and syntax, whereas together with their counterparts, e.g., coactivators/repressors and chromatin modifiers/remodelers, they regulate gene expression [1,16]. Genomic abnormalities within TFs’ coding DNA sequences or/and across their cis-acting transcriptional determinants, e.g., super-enhancers (SEs), predispose or even cause oncogenic phenotypes [1,16,19]. The cancer genome has been exhaustively studied since the advent of omics methodologies that illuminate previously unknown sequence alterations of cis-regulatory DNA elements and dysfunctions of protein molecules, e.g., TFs, that dictate oncogenesis [1]. Such abnormalities lead to oncogenic gene expression programs directed by TFs and other signaling components, such as MYC, KRAS, TAL-1, etc., that have been “crowned” as master oncogenic drivers [16,20,21]. For instance, an extended repertoire of human cancers is linked to quantitative or qualitative diversifications of *MYC*, and many of those are coupled to metabolic adaptation of the neoplastic cellular states [16]. *MYCN* is a member of the *MYC* proto-oncogene family, which also includes *MYC* and *MYCL*, and encodes a basic helix–loop–helix–leucine zipper (bHLH–LZ) TF, initially characterized in neuroblastoma [22,23,24,25]. MYCN oncoprotein encompasses conserved amino acid stretches termed *MYC* boxes, contains a C-terminal domain that serves as a DNA binding domain (DBD), and dimerizes with MAX through its bHLH–LZ domain. MYCN gains access to chromatin landscapes endowed with a consensus E-box [CAC(G/A)TG] or its degenerate DNA binding sites [23] and works as a transcriptional modulator of numerous target genes involved in vital cellular processes, including but not restricted to metabolism [23].

Dysregulated gene expression landmarks the evolution of metabolic dependencies in human tumors, including those established in neuroblastoma, a solid malignant tumor of the sympathetic nervous system. The disease is the most common extracranial cancer in children [26,27], accounting for ~7% of the total cancers in childhood and ~15% of those that lead to children’s deaths [28]. Neuroblastoma patients exhibit various phenotypes that often correlate with the age of diagnosis [29]. They are primarily classified into four groups, as very low-, low-, intermediate-, and high-risk, based on their disease stage (according to the International Neuroblastoma Risk Group Staging System (INRGSS)), their age at the time of diagnosis, their histological type (maturing ganglioneuroma versus ganglioneuroblastoma, intermixed versus ganglioneuroblastoma, or nodular versus neuroblastoma), their grade, their *MYCN* gene status, their 11q chromosomal status, and their tumor cell ploidy [28,30]. Stage L1 refers to tumors located only in the area where they started, L2 tumors are spread both to nearby and other areas, M tumors have spread to other parts of the body, and MS tumors have spread to only the liver, skin, and/or bone marrow in patients younger than 18 months [30].

Based on the INRGSS, very low-risk tumors encompass stage L1/L2 maturing ganglioneuroma or intermixed ganglioneuroblastoma, stage L1 tumors with non-amplified *MYCN*, and stage MS in children younger than 18 months of age with no 11q aberration. Low-risk neuroblastomas include stage L2 in children younger than 18 months of age with no 11q aberration; L2 in children older than 18 months of age with ganglioneuroblastoma, nodular, or differentiated neuroblastoma with no 11q aberration; and stage M in children younger than 18 months without *MYCN* amplification and hyperdiploidy. Intermediate-risk neuroblastomas include stage L2 in children younger than 18 months without *MYCN* amplification but with 11q aberration; stage L2 in patients older than 18 months with ganglioneuroblastoma, nodular, or neuroblastoma with differentiating histology and 11q aberration; stage L2 in patients older than 18 months with ganglioneuroblastoma, nodular, or poorly differentiated or undifferentiated neuroblastoma; stage M in children younger than 12 months with diploidy; and stage M in children 12 months to 18 months old with diploidy. High-risk neuroblastomas include stage L1 and L2 tumors with *MYCN* amplification, stage M in patients younger and older than 18 months of age with *MYCN* amplification, stage MS in children younger than 18 months with 11q aberration, and stage MS in children younger than 18 months of age with *MYCN* amplification [30,31].

Neuroblastoma treatment is designed based on the risk assessment, with most patients with very low- and low-risk tumors undergoing surgery alone, intermediate-risk patients receiving both surgery and chemotherapy (determined by the child’s age, stage, tumor histology, and ploidy, as well as the genetic changes in chromosomes 1p and 11q), and high-risk patients requiring a more intensive treatment including surgery, chemotherapy, radiation, retinoid therapy, immunotherapy, and stem cell transplant [31].

Less than 50% of high-risk patients survive, in contrast to low-risk patients who are cured in the majority of cases [29]. Neuroblastoma tumors exhibit extreme phenotypic heterogeneity that might indicate alternative sensitivity during therapeutic drug applications [32]. Importantly, spontaneous regression of the disease has often been detected in infants [27]. Neuroblastoma cells exhibit divergent metabolic features compared to normal sympathetic neurons [28]. Mechanistically, the disease development is highly connected with, among others, a well-studied genomic abnormality: the amplification of *MYCN* [27,28,29,33], which nowadays has been assigned as a prognostic factor or/and considered as a causal mechanism that drives the disease phenotype evolution [33]. As mentioned above, *MYCN* amplification is frequent in high-risk patients [28] and is utilized for patient-risk classification [34]; however, this is not the only mechanism responsible for the disease, since non-*MYCN*-amplified neuroblastoma is abundantly recorded in patients. Chromosomal abnormalities, such as the gain of 17q, the loss of 1p, and the loss of 11q, have also been associated with worse prognoses [35].

The biology of neuroblastoma and its underlying developmental mechanisms have been progressively elucidated by advanced studies [36,37,38] beyond the context of *MYCN* amplifications that have been mapped both across the endogenous locus and in structures of extrachromosomal DNA amplicons (ecDNA) [1,39]. Recent studies have shown that RAS/MAPK pathway alterations and telomere maintenance mechanisms may also be useful for the discrimination of the high-risk subgroups or for the identification of intermediate-risk subgroups with possible disease recurrence [40,41]. Additionally, mutations in the paired-like homeobox 2B (PHOX2B) and anaplastic lymphoma kinase (ALK) genes have been detected in patients with rare familial neuroblastoma [42,43]. In many cases, neuroblastoma phenotypes are characterized by the aberrant expression of *MYCN* and its downstream interrelationships with critical metabolism-related genes. For example, it has been shown that *MYCN* confers metabolic reprogramming in neuroblastoma cells in cooperation with *ATF4*, and together, they assemble a positive feedback loop which is critical for the activation of the serine–glycine–one carbon (SCOG) biosynthetic pathway in *MYCN*-amplified neuroblastoma cells [44]. In addition, *MYCN* was recently shown to promote de novo lipogenesis in neuroblastoma through direct activation of lipogenic enzymes that orchestrate fatty acid synthesis, a key hallmark of metabolic reprogramming in cancer supporting tumor survival under nutrient deprivation conditions [45]. The engagement of *MYCN* in neuroblastoma metabolic rewiring was further underscored by the finding of its positive feedback loop with enzyme aldehyde dehydrogenase family 18 member A1 (ALDH18A1), a critical component in glutamine metabolism. Increased ALDH18A1 in *MYCN*-amplified neuroblastoma is associated with amino acid and nucleotide metabolism, thus supporting the cell proliferation and self-renewal potency of cancer cells [46].

The role(s) of *MYCN* in metabolic reprogramming-related gene expression program evolution in neuroblastoma has remained largely elusive. This work evaluates the impact of *MYCN* amplification in metabolic reprogramming in neuroblastoma. Two workflows were applied to address this altered metabolic state in the contexts of *MYCN* amplifications and transgenic-based enforced expression of the oncoprotein. We meta-analyzed published datasets to address the impact of *MYCN* overexpression [26,29]. Our findings distinguish a collection of metabolically committed and cancer-related TF-encoding genes that become overexpressed and are “molecularly flagged” by direct *MYCN* binding across their cis-acting regulatory elements. Importantly, comparative analysis of the results reciprocally confirmed our findings and highlighted the functional significance of *MYCN* for neuroblastoma development. Hence, TFs are comprehensively studied at the mechanistic and regulatory level in the context of neuroblastoma metabolism. The emerging new knowledge describes previously unspecified direct targets of MYCN oncoprotein that encode for TFs with regulatory capacities, thus facilitating a better understanding of how genomic alterations drive metabolic phenotypes in cancers.

## 2. Materials and Methods

### 2.1. Transcriptomics Analyses

RNA-seq analyses in neuroblastoma cell lines: Computational meta-analysis comparing *MYCN*-amplified and non-*MYCN*-amplified neuroblastoma cell lines was conducted by utilizing publicly available RNA-seq data described in Boeva et al. [47] (GEO accession number GSE90683). The detailed RNA-seq analysis pipeline and results can be found in Koutsi et al. [1]. In brief, quality control of sequencing reads was conducted using FastQC (Galaxy Version 0.73 + galaxy0). Then, reads were mapped to the hg19 reference human genome using the HISAT2 alignment tool (Galaxy Version 2.2.1 + galaxy0) with “paired-end data from a single interleaved dataset”, “stranded”, and “reverse” parameters [48]. Gene expression quantification was performed using the htseq-count tool with “union mode”, “stranded”, “reverse,” and “minimum alignment quality 10” parameters (Galaxy Version 0.9.1 + galaxy1) [49]. The EdgeR algorithm was employed to identify differentially expressed genes (DEGs) between *MYCN*-amplified and non-*MYCN*-amplified neuroblastoma cell lines with a *p*-value threshold of <0.01 and |log_2_[fold change (FC)]| ≥ 1 considering only the genes with more than ten counts in at least five individual samples. Differentially expressed TFs were identified by utilizing the official list of human TFs [50]. Gene ontology analyses (GOs) of upregulated DEGs (uDEGs) were performed through the gProfiler tool [51]. Selected statistically significant biological processes (*p*-adjusted < 0.05) were visualized as dotplots using the ggplot2 package. gProfiler was selected because it provides information for the experimental or computational annotation origins of the genes assigned to each biological term. This allows the demarcation of metabolism-related genes. Heatmaps of DEGs were constructed using pheatmap and assessing log_2_(CPM) normalized counts.

RNA-seq analyses in neuroblastoma patient-derived samples: (A) We utilized publicly available RNA-seq datasets, described in Rifatbegovic et al. (GEO accession number GSE94035) [52]. The clinical samples were derived from neuroblastoma patients with primary stage 4 metastatic (M) tumors. The total cohort of 16 samples was subjected to rigorous computational processing in order to identify the portion/ratio of immune cells within each sample, thus allowing us to exclude highly heterogeneous datasets from our computational investigation. This step is important to avoid any transcriptional noise that originates from highly “contaminated” samples to dominate our downstream analyses. This was conducted by the application of ABsolute Immune Signal deconvolution (ABIS), an efficient tool for clinical sample profiling that assesses gene expression values and is available at https://giannimonaco.shinyapps.io/ABIS/ (accessed on 13 September 2023) [53]. Six samples that met the criteria and displayed low “contamination” from immune cells were selected for further transcriptomics analysis. Three of those were *MYCN*-amplified and three were non-*MYCN*-amplified and they shared similar-limited enrichment of immune cells (≤10%). RNA-seq analyses were conducted as described above with minor modifications outlined below: Sequencing reads were mapped to the hg19 reference human genome by utilizing the HISAT2 alignment tool (Galaxy Version 2.2.1 + galaxy0) and applying “single-end” and “unstranded” options [48]. The calculation of reads that were mapped across the genes was performed by the application of the htseq-count tool (Galaxy Version 0.9.1 + galaxy1) in union mode with “unstranded” and “minimum alignment quality 10” options [49]. The EdgeR algorithm was employed to identify DEGs between *MYCN*-amplified and non-*MYCN*-amplified neuroblastoma patient-derived samples with a *p*-value threshold of <0.05 and |log_2_(FC)| ≥ 0.6, considering only the genes with more than ten counts in at least two individual samples. (B) Neuroblastoma datasets from the Therapeutically Applicable Research to Generate Effective Treatments (TARGET) project were also assessed to evaluate the effect of *MYCN* amplification in the metabolic rewiring of neuroblastoma [54]. Raw RNA-seq counts of gene expression quantification (*n* = 162) accompanied by the clinical and histological characteristics of the specimens were retrieved from the National Cancer Institute GDC Data Portal. The samples for which information regarding *MYCN* amplification status was not available were excluded from further analysis. In a second layer of classification, 66 primary tumor samples with undifferentiated or poorly differentiated histological status derived from high-risk and stage 4 patients were subjected to ABIS deconvolution of immune cells. Twenty *MYCN*-amplified and twenty-five non-*MYCN*-amplified samples displayed ≤10% enrichment for immune cells and were utilized for differential gene expression analysis through EdgeR (*p*-value < 0.05 and |log2(FC)| ≥ 0.58). Genes with at least ten counts in twelve samples were processed in the analysis.

### 2.2. ChIP-Seq Data Analyses

To investigate the role of *MYCN* in human neuroblastoma, we utilized ChIP-sequencing FASTQ files obtained from the Gene Expression Omnibus database (GEO; https://www.ncbi.nlm.nih.gov/geo/, accessed on 21 June 2023) (GEO accession number GSE138315). Meta-analyses were conducted by integrating publicly available data from two distinct groups of neuroblastoma cell lines based on *MYCN* amplification status. The analysis was conducted using three *MYCN*-amplified neuroblastoma cell lines (*COG-N-415*, *LA-N-5*, and *NB-1643*) and one non-*MYCN*-amplified neuroblastoma cell line (*NB-69*) as a control. Detailed information about these samples is provided in [29]. Furthermore, we utilized publicly available MYCN-ChIP-seq data from the SHEP *MYCN*-ER cell line, in which *MYCN* overexpression is achieved under a 4-hydroxytamoxifen (4-OHT)-activating *MYCN*, while the control cells were treated with DMSO (GEO accession number GSE199086) [26]. Moreover, for monitoring the ASCL1-binding profile in a human *MYCN*-amplified neuroblastoma cell line, we utilized a publicly accessible ASCL1-ChIP-seq FASTQ file from Kelly cells, which was obtained from the GEO database (GEO accession number GSE120074) [55]. ChIP-sequencing data were analyzed by utilizing the Galaxy online platform (https://usegalaxy.org/, accessed on 21 June 2023) [56], a widely used tool for bioinformatics analysis. Initial quality assessment of the sequencing reads was performed using FastQC (Galaxy Version 0.73 + galaxy0). Adaptors and low-quality sequences were trimmed, using Trim Galore (Galaxy Version 0.6.7 + galaxy0) with default parameters. Trimmed reads were then aligned to the hg19 reference human genome using the Bowtie2 tool (Galaxy Version 2.5.0 + galaxy0) with “single-end” or “paired-end” and “very sensitive end-to-end” parameters [57]. Duplicate reads were removed using the RmDup tool (Galaxy Version 2.0.1) from the SAMtools package [58]. To ensure same-scale comparisons, the samples were normalized for their sequencing depth using the Downsample SAM/BAM tool (Galaxy Version 2.18.2.1). Peak calling was performed by MACS2 (Galaxy Version 2.2.7.1 + galaxy0), with the input data serving as control files and default parameters [59]. Peaks were considered significant using a q-value cutoff of 0.05. To ensure data reliability, peaks detected in chromosome Y and unplaced contigs (e.g., chrUn and chrM) as well as those found in hg19 ENCODE blacklisted regions known to have low sequencing assurance were excluded [60]. The reads that mapped within the identified peaks were counted using MultiCovBed (Galaxy Version 2.30.0) [61]. The quantification of the signal intensity was performed using the bamCoverage tool from the deepTools package (Galaxy Version 3.5.1.0.0) with the “normalize to reads per kilobase per million (RPKM)” and “average fragment size = 300 bp” options, which generated bigwig files [62]. For the identification of inducible peaks, a comparative analysis was conducted by calculating the FC values between the *MYCN*-amplified neuroblastoma cell lines (*COG-N-415*, *LA-N-5*, and *NB-1643*) and non-*MYCN*-amplified neuroblastoma cell line (*NB-69*). Similarly, in the case of *MYCN* overexpression [26], inducible peaks were identified according to their FC value between SHEP *MYCN*-ER 4-OHT and SHEP *MYCN*-ER DMSO. We set a minimum FC cutoff of ≥2 and then the inducible peaks were filtered based on a minimum number of reads, specifically, 20 reads for *MYCN*-amplified neuroblastoma cell lines and 40 reads for SHEP *MYCN*-ER 4-OHT. Common MYCN-inducible peaks between the different *MYCN*-amplified neuroblastoma cell lines and/or SHEP *MYCN*-ER 4-OHT were identified using the “Intersect” function of the bx-python package (Version 0.7.1), which was accessed through Galaxy. This analysis allowed us to identify the overlapping inducible peaks that were consistently shaped by MYCN across the different experimental conditions. Additionally, we employed the “Intersect” function (Version 0.7.1) to identify ASCL1 peaks that overlapped with the common MYCN-inducible peaks across the different *MYCN*-amplified neuroblastoma cell lines and SHEP *MYCN*-ER 4-OHT over-expressing system. Furthermore, the assignment of ChIP-seq peaks to the nearest transcription start site (TSS) of the hg19 reference human genome was determined by the Genomic Regions Enrichment of Annotations Tool (GREAT) (version 4.0.4), employing the whole genome as background and the “single nearest gene” parameter within 1000 kb [63]. In addition, the identification and analysis of transcription factor binding motifs (TFBMs) within the ChIP-seq peaks were carried out using the MEME-ChIP [64] and AME (Analysis of Motif Enrichment) [65] tools, with motifs from the JASPAR 2022 CORE non-redundant vertebrate database as inputs (http://jaspar.genereg.net, accessed on 22 June 2023). For MEME-ChIP, the options “DNA -mod zoops or -mod anr -minw 4 -maxw 15” were applied, and for AME, the options “-scoring average -method fisher—hit-lo-fraction 0.25—control shuffle” were used to identify enriched motifs within the ChIP-seq peaks. In both MEME-ChIP and AME analyses, an E-value threshold for motif enrichment and a *p*-value cutoff of < 0.05 for statistical significance were considered, ensuring the identification of TFBMs that exhibited biological relevance and statistical significance within the ChIP-seq peaks. Furthermore, the identification of E-boxes was centered on the inducible peaks located in close proximity to *BCL6*, *DLX1*, *NRF2*, *ATF4*, and *STAT5B* genes. To conduct this analysis, we utilized the MAST (Motif Alignment and Search Tool) and FIMO (Find Individual Motif Occurrences) tools, accessed through the MEME-Suite [64,66,67], applying default parameters. The E-box motifs used as inputs for scanning were sourced from the JASPAR 2022 CORE non-redundant vertebrate database (MA0059.1 and MA0819.2) [68]. The MAST tool was also utilized for the computational analysis of TFBMs recognized by KLF4, STAT5B, STAT5A::STAT5B, and BCL6 across the *MYCN* locus. Specifically, the relevant TFBMs used as inputs for this scanning were sourced from the JASPAR 2022 CORE non-redundant vertebrate database (MA0039.4, MA1625.1, MA0519.1, and MA0463.2). Finally, the visualization and exploration of signals in specific genomic regions were performed using the IGV (Integrative Genomics Viewer version 2.16.1) browser [69].

## 3. Results

### 3.1. Gene Expression Rewiring and Metabolic Signatures in MYCN-Amplified Neuroblastoma

**Figure 1:**
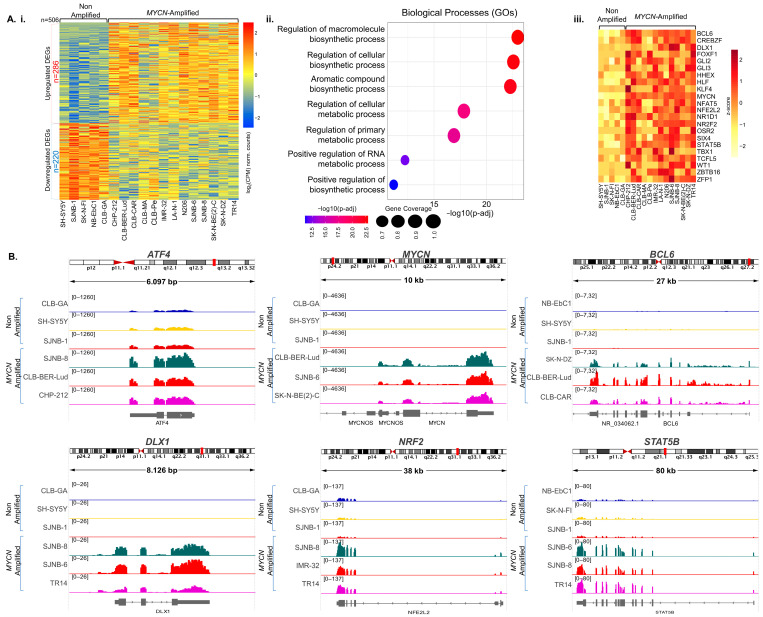
Transcriptional profiling of *MYCN*-amplified and non-amplified cell lines. (**A**): (**i**) RNA-seq heatmap depicting the full spectrum of 506 differentially expressed genes (DEGs) in *MYCN*-amplified compared to non-*MYCN*-amplified cell lines. Of those DEGs, 286 are upregulated (uDEGs) and 220 are downregulated. The heatmap was generated using log_2_(CPM) normalized counts. (**ii**) Selected examples of the most statistically and biologically significant biological processes associated with metabolism-related transcription factors (TFs), as determined by gene ontology analyses (GOs). (**iii**) RNA-seq heatmap illustrating the transcriptional upregulation of the 22 renowned metabolism-related TFs. log_2_(CPM) normalized counts were used for visualization. (**B**) High-resolution Integrative Genomic Viewer (IGV) snapshots of transcriptomics signals, sharply illustrating RNA-seq signal intensities across the genomic loci (exons) of selected uDEGs encoding for metabolism-related TFs (*ATF4*, *BCL6*, *DLX1*, *NRF2*, *STAT5B*). The *MYCN* exhibits strong RNA-seq signal intensities in *MYCN*-amplified cell lines, as anticipated, thus validating the specificity and sensitivity of our analysis. Two subsets composed of three out of the five non-*MYCN*-amplified and three out of the thirteen *MYCN*-amplified cell lines were selected for each individual locus visualization.

To monitor the gene expression in human neuroblastoma and address the impact of *MYCN* amplification in in vivo metabolic reconstitution, we utilized our established workflow that was previously applied [1] for intratumoral computational transcriptional profiling in neuroblastoma published results [47]. We applied stringent criteria to avoid any transcriptional noise dominating our downstream analyses, according to published strategies [70]. The results indicated the profile of activation and repression of DEGs (FC ≥ 2; *p*-value < 0.01), which then were evaluated according to the function of their encoded products (Figure 1(Ai)). We fished out 286 uDEGs in *MYCN*-amplified compared to non-*MYCN*-amplified neuroblastoma cell lines and processed them through GOs (Supplementary Figure S1A). Next, we distinguished 38 that encode for TFs according to the official TF list [50] (Table S1). At this stage, the functional significance of those TFs in metabolism was assessed primarily based on their classification to specific processes via GOs (Supplementary Figure S1A and Figure 1(Aii)). We selected 36 TFs that have been previously recorded in metabolism-related regulatory functions (Supplementary Figure S1B). In particular, GOs highlighted metabolic pathways such as the cellular metabolic process and macromolecule biosynthetic process (Figure 1(Aii)). This was accompanied by evaluation of the TFs’ function based on published evidence, which resulted in 22 TFs, including, among others, *DLX1*, *KLF4*, *NRF2*, *BCL6*, *STAT5B*, and *MYCN* (Figure 1(Aiii)). In addition, manual curation highlighted *ATF4* upregulation in *MYCN*-amplified compared to non-*MYCN*-amplified neuroblastoma cell lines, even with less induction (FC 1.77, *p*-value 0.047) (Supplementary Figure S1B). Integrative Genomic Viewer analyses (IGVs) display selected examples of those findings (Figure 1B). Thus, the above integrative analyses captured metabolism-related TF upregulation in neuroblastoma cell lines that bear copy number genomic variations of *MYCN*.

#### 3.1.1. *MYCN*-Binding Landscape in Neuroblastoma Illuminating Novel TF-Encoding Target-Genes

**Figure 2:**
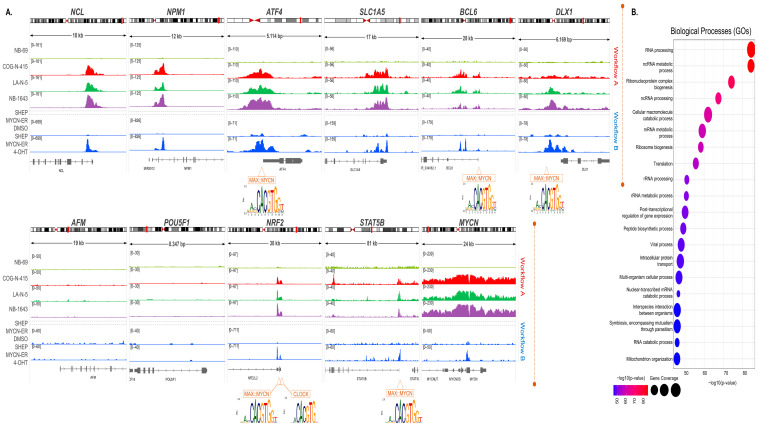
The MYCN cistrome in neuroblastoma. (**A**) High-resolution IGV snapshots display MYCN binding in chromatin landscapes proximal to the TSSs of known MYCN target genes (*ATF4*, *NCL*, *NPM1*, *SLC1A5*), MYCN off-targets [*AFM*, *OCT4* (*POU5F1*)], upregulated genes that encode for metabolism-related TFs (*BCL6*, *DLX1*, *NRF2*, *STAT5B*), and the endogenous *MYCN* locus. Strong inducible MYCN binding is illustrated in both workflows of examination, assessing *MYCN*-amplified and non-*MYCN*-amplified cells (workflow A) and the SHEP *MYCN*-ER 4-OHT overexpressing system (workflow B). *ATF4*, *BCL6*, *DLX1*, *NRF2*, and *STAT5B* transcription start site (TSS)-proximal genomic loci harbor E-boxes recognized by MYCN::MAX or CLOCK, legible by MYCN oncoprotein. Of significant interest is the difference observed in MYCN binding between *MYCN*-amplified cell lines and the *MYCN*-overexpressing system of study (SHEP *MYCN*-ER 4-OHT). In *MYCN*-amplified cell lines, tandemly arranged *MYCN* amplifications, spanning several kbs, provide an expanded chromatin landscape, allowing for broad MYCN binding. In contrast, in the *MYCN*-overexpressing system, a precisely shaped peak of approximately 1 kb length is mapped across the TSS-proximal locus. (**B**) GOs, applied on the full spectrum of 7971 common inducible MYCN-ChIP-seq peaks obtained from both workflows of our study, highlight the specificity of MYCN-inducible binding for metabolism-related functions including RNA processing, macromolecule biosynthesis, peptide biosynthesis, etc.

To investigate how *MYCN* amplification mechanistically imposes the metabolic rewiring of neuroblastoma cells, we first attended to the in cis distribution of its encoded product (Figure 2A upper and lower panels). MYCN binding was monitored at the whole chromosomal and regional scales. We utilized published ChIP-seq datasets (workflow A) and conducted genome topology assessments from the ground state (see Materials and Methods). We applied our in-house workflow; published ChIP-seq datasets obtained from studies in human neuroblastoma cell lines, which carried *(COG-N-415, LA-N-5,* and *NB-1643)* or lacked *(NB-69)* genomic amplifications of the *MYCN* as described [29], were subjected to rigorous bioinformatics processing (see Materials and Methods). Initially, MYCN-ChIP-seq peaks were called, followed by individual pairwise comparisons of their signals’ intensities implemented between *NB-69* and *COG-N-415*, *LA-N-5*, and *NB-1643*. A list of inducible signals was generated for each assessment (FC ≥ 2; enriched in *MYCN*-amplified cell lines compared to non-*MYCN*-amplified) and showed 32,749 for *COG-N-415*/*NB-69*, 38,777 for *LA-N-5*/*NB-69*, and 30,809 for *NB-1643*/*NB-69* comparisons, respectively (Table S2). Next, a step of evaluation of our ChIP-seq meta-analyses was oriented to validate MYCN-binding events across known target loci where the oncogenic TF was captured [71], as well as additional off-targets; this process serves as an internal control. For instance, robust MYCN binding was mapped across the *SLC1A5* (*ASCT2*) locus, which encodes a known regulator of glutamine metabolism, a key feature of neuroblastoma tumorigenesis [71,72] (Figure 2A upper panel). In addition, MYCN binds to the *NCL* locus that encodes the nuclear RNA-binding protein nucleolin, essential for rRNA processing and ribosome biogenesis [26,73], and the *NPM1* locus which encodes the multifunctional nuclear protein nucleophosmin, known for ribosome biogenesis and genome stability regulation (Figure 2A upper panel). In sharp contrast, off-target loci (e.g., *AFM*, *OCT4*) were scanned and demonstrated as inattentive/not significant in MYCN binding (Figure 2A lower panel). IGVs sharply display the above findings in high-resolution, thus ensuring the validity of our approach. In another layer of evaluation, we detected MYCN binding across the *ATF4* locus, an anticipated result since the *MYCN*–*ATF4* axis is one of the well-addressed TF interrelationships in neuroblastoma [28,44]. This established feedback loop is crucial for increasing the amino acid availability and, consequently, sustaining *MYCN*-mediated growth in neuroblastoma cells. Thus, *MYCN* collaborates with *ATF4* in regulating *ASCT2* expression, but in addition, it targets the *ATF4* cis-acting regulatory region (IGVs) (Figure 2A upper panel). The above results authenticate both the novelty and the accuracy of our approach.

Next, we examined the cistrome of MYCN across chromatin landscapes that host genomic loci that encode for several of the upregulated metabolism-related TFs demarcated above. We zoomed in across the chromatin microenvironments of residence and monitored the patterns of MYCN genomic distribution. IGVs depict in high-resolution that the *BCL6* locus hosts enhanced MYCN binding, predominantly enriched ~5 kb downstream of the TSS and at a lesser but significant intensity on the promoter of the gene (Figure 2A upper panel). Indicatively, precisely shaped ChIP-seq signals are mounted across these vicinities. These binding profiles are mapped in all three *MYCN*-amplified, but not in the non-*MYCN*-amplified, neuroblastoma cell lines. Transcription factor binding motif analyses (TFBMs) demonstrated that MYCN masks regulatory sequences enriched for TF binding sites (TFBSs), including E-boxes recognized from MYCN::MAX (Figure 2A upper panel), as evaluated by both MAST tool- and FIMO-based analyses [64,66,67]. Interestingly, these genomic coordinates reconstitute in vivo chromatin landscapes that coincide with the well-addressed SE of the gene, described in other types of cancers [19]. Hence, *BCL6* is tagged by MYCN binding, a profile that mechanistically is in line with its transcriptional upregulation in *MYCN*-amplified neuroblastoma cells. *DLX1* hosts enhanced MYCN binding, predominantly enriched across a DNA stretch of ~1 kb that flanks its TSS and encompasses the core promoter, as well as TFBSs for FOXM1 that were previously characterized in ovarian cancer [74] (Figure 2A upper panel). Indicatively, precisely shaped ChIP-seq signals are mounted across these vicinities. These binding profiles are mapped in all three *MYCN*-amplified (more intensively in *LA-N-5* and *COG-N-415*), but not in non-*MYCN*-amplified, neuroblastoma cell lines. TFBM analyses demonstrated that MYCN masks regulatory sequences enriched for TFBSs, including E-boxes recognized from MYCN::MAX, as evaluated by both MAST tool- and FIMO-based analyses. Hence, *DLX1* is tagged by MYCN binding, a profile that mechanistically is in line with its transcriptional upregulation in *MYCN*-amplified neuroblastoma cells. The *NRF2* locus hosts enhanced MYCN binding, predominantly enriched across a DNA stretch of ~2 kb that flanks its TSS and encompasses the core promoter. Indicatively, precisely shaped ChIP-seq signals are mounted across these vicinities (Figure 2A lower panel). These binding profiles are mapped in all three *MYCN*-amplified, but not in non-*MYCN*-amplified, neuroblastoma cell lines. TFBM analyses demonstrated that MYCN masks regulatory sequences enriched for TFBSs, including E-boxes recognized from MYCN::MAX (Figure 2A lower panel), as evaluated by both MAST tool- and FIMO-based analyses. These results illuminate that *NRF2* is a direct target of *MYCN*, thus suggesting their regulatory interrelationship in neuroblastoma. The *STAT5B* locus hosts enhanced MYCN binding, predominantly enriched across a DNA stretch of ~0.7 kb that flanks its TSS and encompasses the core promoter. Indicatively, precisely shaped ChIP-seq signals are mounted across these vicinities. These binding profiles are mapped in all three *MYCN*-amplified, but not in non-*MYCN*-amplified, neuroblastoma cell lines. In addition, *COG-N-415* cells exhibit another intragenic MYCN-binding signal that masks introns and exons of *STAT5B*. TFBM analyses demonstrated that *MYCN* masks regulatory sequences enriched for TFBSs, including E-boxes recognized from MYCN::MAX (Figure 2A lower panel), as evaluated by both MAST tool- and FIMO-based analyses. These results illuminate that *STAT5B* is a direct target of *MYCN*, thus suggesting their regulatory interrelationship in neuroblastoma.

Evidently, our results underscore that *MYCN* is substantially committed to metabolic rewiring in neuroblastoma cells through its attachment to transcriptional regulatory cis-elements that neighbor metabolism-related TF-encoding genes. To gain further mechanistic confirmation, we applied the same workflow to an alternative system of study that relies on the enforced expression of *MYCN* (workflow B). More specifically, Wang et al. [26] conducted an advanced study by utilizing the SHEP *MYCN*-ER cell line [34], which bears a single copy of a 4-hydroxytamoxifen (4-OHT)-inducible *MYCN* transgene. This allows for a fine-tuned overexpression of *MYCN* based on the addition of 4-OHT. SHEP *MYCN*-ER cells treated with DMSO were utilized as a control. MYCN-ChIP-seq datasets were analyzed according to our in-house protocol described above and subjected to rigorous bioinformatics processing (see Materials and Methods). In brief, MYCN-ChIP-seq peaks were called, followed by pairwise comparison of their signal intensities between 4-OHT-treated and DMSO-treated SHEP *MYCN*-ER experiments. A list of 11,308 inducible signals was generated (FC ≥ 2; enriched in 4-OHT-treated cells compared to DMSO-treated cells) (Table S2). Next, we monitored in high-resolution the distribution of these signals across the loci that host known- and off-targets, described above, and we verified to a significant extent the results in both systems, thus confirming the specificity of our findings (Figure 2A upper and lower panels). IGVs substantially validate the above findings, display in high-resolution MYCN-binding profiles that match those obtained from the above analyses, and demonstrate at the genomic loci-specific level that TSS-proximal cis-acting elements of the genes of investigation are occupied by MYCN in both systems of study. Overall, the binding profiles shaped recapitulate those that emerged from the analyses of the *MYCN*-amplified versus non-*MYCN*-amplified neuroblastoma cells and depict robust binding of the MYCN oncoprotein upstream of or flanking the TSSs of the above target-genes and control loci (Figure 2A upper and lower panels). Indeed, these patterns of genomic distributions of MYCN assembled during its attachment to the respective chromatin landscapes, in both cell systems of study, are overlapping or nearly identical. Moreover, our computational investigation was complemented with another layer of evaluation; we assessed the endogenous *MYCN* for MYCN binding and identified that (a) *MYCN* exhibits auto-regulatory capabilities facilitated by direct targeting of its own coding locus by its encoded oncoprotein. This phenomenon is evident in both systems of study. This is consistent with previous studies that mapped MYCN binding across extended genomic loci that harbor *MYCN* in neuroblastoma [75]. Interestingly, it has been shown that this type of autoregulation can be disrupted in amplified, but not in single-copy, neuroblastoma cell lines [76]. This underscores the impact of genomic amplification in the establishment of diverse mechanistic rules for the same disease. (b) Furthermore, our results show that in neuroblastoma cell lines that carry (*COG-N-415*, *LA-N-5*, and *NB-1643*) genomic amplifications of *MYCN*, robust binding of the TF is mapped across an extended (≥10 kb) genomic region. This pattern is inattentive/not significant, or even abolished, from the non-*MYCN*-amplified cells (*NB-69*). Given that *MYCN* amplification routinely spans several or even hundreds of kb in cis [77,78], the multiple copies generated can first provide an amplified chromatin landscape that serves as an anchored site for MYCN binding and, in addition, can multiply intact amplicons of coding genes resulting in overexpression of MYCN oncoprotein. Thus, it is realistic to argue that *MYCN* amplifications footprint the endogenous loci at the (epi)genomic level. (c) On the other hand, in the SHEP *MYCN*-ER cell line system of study, 4-OHT-inducible binding of MYCN is centered around the TSS-proximal intronic and exonic sequences of *MYCN* and mounts a precisely shaped peak that masks a DNA stretch of ~1 kb. This sounds realistic since this cell line harbors a single copy *MYCN* transgene and lacks *MYCN* amplicons. The above results imply that in both systems an auto-regulatory mechanism facilitates *MYCN* overexpression with or without affecting the (epi)genome architecture of its residential chromosomal coordinates. This is made more intriguing when considering that outside of chromosomal landscapes *MYCN*-ecDNA-amplicons still work and encode *MYCN* efficiently. Finally, an additional layer of verification was achieved by the intersection of the individual spectrums of the inducible MYCN peaks that emerged from the whole meta-analysis. We sequentially overlayed the individual spectrums of MYCN-ChIP-seq inducible peaks following a gradual decreasing mode of analysis. The results depicted 7971 common signal instances, which then were subjected to GOs (Figure 2B). The outcome of this comparative assessment sharply illustrates strong correlation between the genomic distribution of MYCN oncoprotein with metabolism-related processes in neuroblastoma cells (Figure 2B). More specifically, GOs highlighted, among others, cellular functions related to RNA metabolism, cellular macromolecule catabolic processes, ribonucleoprotein complex and ribosome biogenesis, peptide biosynthetic processes, etc. Given that those genomic loci are randomly distributed across chromosomal landscapes and that they were ChIPed through MYCN binding, a clear correlation of the TF with metabolic rewiring is underscored. This suggests the assembly and function of an *MYCN* axis that involves additional metabolism-related TFs, such as those comprehensively discussed above and in the discussion section. The pleiotropic commitment of those regulators of gene expression in vital cellular processes implies the in vivo reconstitution of a newly evolved metabolic state in neuroblastoma.

**Figure 3:**
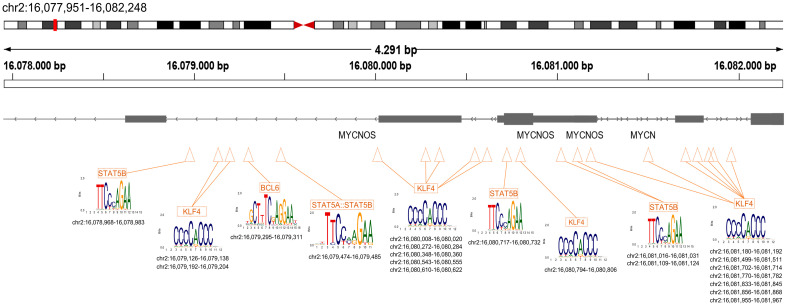
Computational analysis of transcription factor binding motifs (TFBMs) across *MYCN*. The MAST-tool assessed a genomic region of ~4.3 kb that flanks the TSS of *MYCN*. TFBMs recognized by KLF4, STAT5B, and BCL6 are embedded with the genomic loci that harbor *MYCN*. The genomic coordinates are highlighted at the bottom of the figure.

Moreover, we investigated the enrichment of the *MYCN* genomic locus for TFBMs recognized by the above TFs. We applied MAST-tool-based analysis and processed a genomic region of ~4.3 kb that flanks the TSS of *MYCN* (chr2:16,077,951–16,082,248) (Figure 3). We mapped several motifs for KLF4, multiple for STAT5B, and a single one for BCL6, randomly distributed across this DNA sequence. This profile poises for the binding of those MYCN-targeted TFs across *MYCN*. Given that *MYCN* amplifications result in the in cis expansion of additional copies of the amplified DNA sequence, the above TFBMs are anticipated to become multiplied. Hence, the possibility of the assembly of an auto-regulatory loop between those transcriptional regulatory proteins and *MYCN* cannot be excluded. This is in line with the biology of TFs’ expression regulation, per se. Finally, we investigated the binding profile of ASCL1, a basic helix–loop–helix TF that is expressed in neuroblastoma cells and is required for cell growth and arrest of differentiation [55]. *ASCL1* cis-regulatory elements are targeted by MYCN and additional TF members of the adrenergic (ADRN) neuroblastoma core regulatory circuitry (CRC) [55]. We meta-analyzed one ASCL1-ChIP-seq dataset derived from experiments in Kelly cells and an *MYCN*-amplified neuroblastoma cell line, described in [55]. First, we called 10,661 ASCL1-ChIP-seq peaks and intersected them with the common 7971 MYCN-ChIP-seq-inducible peaks, described above (Supplementary Figure S2A). We retrieved 2081 common peaks, and we processed those in GOs. The results show statistically and biologically significant metabolic processes, such as ncRNA metabolic processes, cellular macromolecule metabolic processes, ribonucleoprotein complex biogenesis, etc. (Supplementary Figure S2A). Next, we monitored the profile of ASCL1 binding across several genomic loci, including *MYCN* and other upregulated metabolism-related TFs, described above. The results highlight proximal binding events of ASCL1 in many of those genes, including *KLF4*, *NRF2*, *GLI2*, and *MYCN* (Supplementary Figure S2B). This analysis strengthens our findings, since it illuminates that several of the upregulated metabolism-related TFs described in our computational study are targeted by an additional TF that is critical for neuroblastoma and is also an *MYCN* target. *ASCL1* was captured enriched, but not statistically significant upregulated, in our transcriptomics meta-analysis between *MYCN*-amplified and non-*MYCN*-amplified cell lines, but this cannot exclude the possibility for a regulatory role in fine-tuning the transcription of several of the upregulated metabolism-related TFs genes discussed in our work. These findings open new avenues for further investigations in the neuroblastoma field.

#### 3.1.2. Computational Validation in Neuroblastoma Patient-Derived Samples

To further confirm our findings and to gain additional insights into the biology of the disease, we assessed gene expression profiles in clinical samples derived from neuroblastoma patients. Transcriptomics profiling in patient-derived surgically removed solid tumors frequently suffers from intrinsic tumor heterogeneity and/or enrichment of the specimens with irrelevant cells, e.g., blood/immune cells, stroma cells, etc. These matter against the precise recording of any differentiation between the transcriptomes of individual samples [79]. In cases of TF investigations, this becomes more challenging, since they are broadly expressed in multitudes of tissue types and, consequently, the monitoring of the expression states of their coding genes, in such systems of study, can be hindered when “non-cancerous”/irrelevant cell types are significantly represented within the samples of examination. Those drawbacks can lead to the domination of the entire computational analyses by “background” signals. To bypass these barriers, we applied a rigorous computational rationale that significantly facilitates the abolishment of “non-cancerous”-derived/non-specific RNA-seq signals yet maintains the balance between the samples and the sensitivity of the analyses (see Materials and Methods). We utilized publicly available RNA-seq clinical datasets and performed transcriptomics meta-analyses centered on both the metabolic rewiring and the 22 metabolism-related TFs identified. First, we curated datasets from stage 4/M metastatic neuroblastoma patients [52] and processed them, with emphasis on the deconvolution from immune cells, by the application of the ABIS tool [53]. From the entire pool of sixteen samples evaluated, six met the applied criteria, and were meticulously selected for further investigation. An equal number of datasets derived from patient samples from both categories (three *MYCN*-amplified neuroblastoma and three non-*MYCN*-amplified neuroblastoma) that are characterized by similar-low enrichment for immune cells (≤10%) were comparatively studied. The results depicted 2726 DEGs [|log_2_(FC)| ≥ 0.6 and *p*-value < 0.05] between these groups. Of those, 1396 DEGs were upregulated (Table S3). GOs applied on the 1396 uDEGs highlighted, among others, several ontologies and functions connected to metabolism, including peptide biosynthetic, amide metabolic, and cellular macromolecule processes, etc. (Supplementary Figure S3(Ai)). Next, we intersected the spectrum of 1396 uDEGs with the one of 286 uDEGs initially identified in cell lines and revealed 77 common genes (Table S3). Of those, eight encode for metabolism-related TFs included in the pool of the 22 TFs initially identified, such as *MYCN*, *BCL6*, *KLF4*, *SIX4*, *NR2F2*, *GLI2*, *GLI3*, and *WT1*. Two additional metabolism-related TF-encoding genes, *ATF4* and *DLX1*, were captured that were commonly upregulated according to FC but not statistically significant according to the *p*-value threshold and were thus excluded from the heatmap (Supplementary Figure S3(Aii)). These findings significantly validate the knowledge derived from the studies on cell lines.

Next, we explored another cohort of samples derived from neuroblastoma patients annotated in TARGET-NBL (neuroblastoma project) [54]. Rigorous criteria were applied and gated the samples that derived from primary, undifferentiated or poorly differentiated, high-risk, and stage 4 neuroblastoma tumors. The pool of 66 samples was then processed for deconvolution of absolute immune signals, as described above, and resulted in a sub-cohort of 45 samples composed of 20 *MYCN*-amplified and 25 non-*MYCN*-amplified neuroblastoma tumors that are characterized by similar-low enrichment from immune cells (≤10%). Comparative transcriptomics analysis highlighted 3424 uDEGs (Table S3), and GOs revealed, among others, cellular biosynthetic, peptide metabolic, and rRNA metabolic processes (Supplementary Figure S3(Bi)). Next, we intersected the spectrums of 3424 uDEGs and 286 uDEGs and identified 110 common genes. Of those, ten encode for metabolism-related TFs included in the pool of the 22 TFs initially identified, such as *FOXF1*, *OSR2*, *WT1*, *MYCN*, *NR2F2*, *SIX4*, *GLI2*, *NR1D1*, *HLF*, and *CREBZF*. Interestingly, manual curation of *ATF4* depicts a statistically significant *p*-value threshold even with lower induction (FC 1.42), whereas *DLX1* was captured while upregulated according to its FC but was not statistically significant according to the *p*-value threshold; thus, both were excluded from the heatmap (Supplementary Figure S3(Bii)). In line with the results described above, these findings also validate the knowledge emerging from the studies on cell lines. Overall, our findings illuminate previously unspecified elements of the complex transcriptional regulatory landscape(s) assembled and underscore the essential commitment of TFs during the metabolic rewiring occurring in neuroblastoma.

## 4. Discussion

Human nervous system metabolism has been elucidated both under normal and oncogenic states [3]. Herein, we conducted alternative workflows and succeeded in addressing the interrelationship of several TFs with *MYCN* overexpression, in neuroblastoma. Among them, *BCL6*, *KLF4*, *DLX1*, *NRF2*, and *STAT5B* are prominent and involved in metabolic regulation. In more detail, the *BCL6* gene, also known as B-Cell Lymphoma 6, resides at Chromosome 3q27.3 in a genomic region of 24.3 kb and encodes a TF that is a member of the Broad-Complex, Tramtrack, and Bric-a-Brac/Pox Virus and Zinc Finger (BTB/POZ) family [80,81]. Ιt was initially identified as a genomic region susceptible to chromosomal abnormalities in cases involving the diffuse large B-cell lymphoma (DLBCL) family [80] and follicular lymphoma (FL) [19,82,83]. BCL6 is a transcriptional repressor that binds DNA through six zinc fingers and can interact with several other factors in order to regulate transcription. It is crucial for B cell development and overexpressed in lymphomas, while it also serves as a diagnostic and prognostic marker, facilitating patient stratification and treatment decision-making [84,85]. Mutations which disrupt two neighboring binding sites that are recognized by transcriptional repressors of the gene diminish the capacity of BCL6 to effectively bind to its own promoter, thereby disrupting the negative autoregulatory mechanism that maintains control over the expression levels of *BCL6* [81,86]. In addition, an intragenic super-enhancer (iSE) of approximately 33 kb, which spans the first intron of *BCL6*, exhibits a high frequency of mutations in primary DLBCL cases [1,19]. In addition, knock-in mouse models with constitutive expression of *BCL6* in B cells, which resemble the 3q27 chromosomal translocation of the human DLBCL, have been studied. More specifically, Cattoretti et al. [87] showed that mice developed increased GC formation and lymphomas that closely resembled human DLBCL. These findings highlight the critical role of *BCL6* dysregulation in DLBCL [87]. Metabolic reprogramming of B cells has been shown to integrate epigenetic activation of *BCL6* gene to induce germinal center B cell differentiation, thus confirming its crucial pathogenic role in lymphomagenesis and immune diseases [88]. Indeed, *BCL6* has been involved in the pathogenesis of B-acute lymphoblastic leukemia (B-ALL), chronic myeloid leukemia (CML), breast cancer, and non-small cell lung cancer (NSCLC) [85,89,90,91], and thus its dysregulated gene expression is an attractive target for therapeutic interventions. Furthermore, the balance between *MYC* and *BCL6* expression in pre-B-ALL has been previously suggested as a key determinant of cell survival [92].

Notably, *BCL6* has also been proposed to act as a metabolic switch in mouse liver cells, regulating genes involved in lipid metabolism. It functions as a repressor of genes involved in fat burning, being a negative regulator of oxidative metabolism and a promising target for fatty liver disease [93]. Novel compounds, including peptide and small molecule inhibitors, have been developed to target BCL6 effectively and disrupt its interaction with co-repressor complexes, counteracting the effects on its target genes [85].

In neuroblastoma cell lines, differential *BCL6* mRNA expression has been recorded between neuroblastic (N-type) and Schwannian stromal (S-type) cells, being higher in S-type cells [32]. However, *BCL6* expression in the neuroblastic regions of neuroblastoma tumors was associated with enhanced relapse time and increased overall patient survival compared to regions without *BCL6* expression. This positive association of *BCL6* with clinical outcome has also been observed in primary central nervous system lymphoma, follicular lymphoma, and DLBCL, possibly because *BCL6* enhances chemotherapy responses. Although detailed mechanistic studies on the role of *BCL6* expression in neuroblastoma are missing, it can be speculated that, analogous to lymphomas, *BCL6* can mediate the arrest of neural crest cell differentiation involved in tumor development, as well as potentially contribute to the genetic instability observed in neuroblastomas, possibly due to attenuation of the DNA damage response [32].

In turn, the *KLF4* gene, also known as Krüppel-like factor 4, resides at Chromosome 9q31.2 in a genomic region of 4.9 kb. *KLF4* encodes for a DNA binding and zinc finger-containing TF that functions as a master regulator of the reprogramming of somatic cells to induced pluripotent stem cells (iPSCs) along with OCT4, SOX2, and c-MYC (Yamanaka factors) [94]. *KLF4*′s unique structural features confer pioneer regulatory activities: its three zinc fingers bind to a 10 bp cognate DNA sequence (5′-GAGGCGTGGC-3′) [95] and, in addition, it exhibits a low complexity surface efficient in interacting with chromatin remodelers and TFs [96]. *KLF4* functions either as a tumor suppressor or as an oncogene in a tissue-specific fashion. For instance, it has been identified as overexpressed in early breast cancer and squamous cell carcinomas [97]. Oppositely, *KLF4* loss in early colorectal cancers due to targeted proteasome-dependent degradation designates its function as a tumor-suppressor through inhibiting tumorigenesis via regulation of cell-cycle arrest [98]. Regarding its commitment to tumor metabolic rewiring, in breast cancer, elevated *KLF4* expression has been detected and contributes to activating phosphofructokinase (PFKP), a key enzyme involved in the glycolysis pathway [99]. Conversely, in pancreatic cancer, *KLF4* executes an alternative role and inhibits tumor progression by transcriptionally repressing the lactate dehydrogenase (LDHA) enzyme. This operation restricts the glycolysis-dependent altered metabolic reprogramming of cancer cells, suggesting a potential tumor-suppressive function for *KLF4* in this context [100]. In a recent study, a novel interaction between monomethyltransferase *SET8* and *KLF4* was identified. The *SET8*/*KLF4* complex formation negatively affects the transcriptional activation of *SIRT4* by *KLF4*, thus promoting the proliferation-favorable Warburg effect of hepatocellular carcinoma cells [101]. In glioblastoma, *KLF4* methylation-dependent transcriptional activation of UDP-glucose 6-dehydrogenase (*UGDH*) increases the synthesis of glycosaminoglycans (GAGs), which constitute essential structural polymers/components of the extracellular matrix (ECM), thus facilitating tumor growth and invasion [102]. Interestingly, *KLF4* has been described as a putative *MYCN*-target gene [103].

In neuroblastoma tumors, low expression of *KLF4* has been associated with poor survival of patients, while its overexpression in neuroblastoma cell line SH-SY-5Y was shown to inhibit cell proliferation and induce cell arrest by upregulating the cell cycle-inhibitor p21Waf1/Cip1 as well as activating cell differentiation [104]. In our study, many neuroblastoma cell lines with *MYCN* amplification exhibited increased expression of *KLF4*, indicating a potential contribution to the cell cycle dysregulation and the transformation process underlying neuroblastoma development.

The *DLX1* gene, also known as Distal-Less Homeobox 1, resides at Chromosome 2q31.1 in a genomic region of 4.1 kb. *DLX1* encodes for a homeobox-containing TF, a homolog of the renowned TF that is encoded by the *Distal-less* (*Dll*) gene in *Drosophila melanogaster*. *Dll* controls the development of limb appendages, such as the distal part of the leg, the assembly of structures of the peripheral nervous system, etc., in fruit flies [105,106,107,108,109]. *DLX1* in humans exerts crucial roles in the assembly of craniofacial structures, and its dysregulation is engaged in distinct types of cancer [110]. For instance, *DLX1* is a reliable biomarker for prostate cancer (PCa), and its elevated expression accompanies the development of aggressive phenotypes of the disease and poor patient survival [111,112]. At the molecular level, DLX1 establishes protein–protein interactions with beta-catenin and activates beta-catenin/TCF signaling pathways, thus promoting growth, migration, and cell colony assembly in Pca [112]. In addition, in normal hematopoiesis, *DLX1* is expressed in a lineage-specific manner, interacts with SMAD4 via its homeodomain, and inhibits *BMP-4*-induced transcriptional activation [110,113]. In aberrant hematopoiesis, such as in acute myeloid leukemia (AML), upregulated expression of *DLX1* has been recorded as a downstream effect of activating mutations of fms-like tyrosine kinase 3 (*FLT3*), a frequent genomic alteration (~30%) that accompanies AML phenotypes [114,115]. Mechanistically, this is served by MAPK and JNK pathways [114]. Finally, in ovarian cancer, *DLX1* is a direct target of Forkhead Box M1 (FOXM1) TF. FOXM1 recognizes two conserved binding sites that flank the TSS of *DLX1*, thus upregulating its expression in high-grade ovarian cancer [74]. Consequently, *DLX1* upregulation promotes cell proliferation, migration, and metastasis. The above findings imply a pleiotropic commitment of *DLX1* in various mechanisms of tumorigenesis. Although there are no studies available elucidating the expression and function of *DLX1* in neuroblastoma, a possible role in the regulation of neural crest cell differentiation mechanisms cannot be excluded, given its expression in neural crest cells [116].

Furthermore, the *NRF2* gene, also known as Nuclear Factor Erythroid 2-related factor 2 (*NFE2L2*), resides at Chromosome 2q31.2 in a genomic region of 34.8 kb. It encodes for a TF that is a cap’n’collar (CNC) member of a family of basic leucine zipper proteins (bZIP) and is composed of 7 Neh domains [117]. *NRF2* can be activated by toxic electrophilic stimuli and oxidant factors to induce the transcription of antioxidant and detoxifying genes, but also genes involved in immune and inflammatory responses, tissue remodeling, and carcinogenesis [118]. However, the primary function of *NRF2* is cell adaptation to stress [119]. It is well known that oxidative stress and metabolic dysfunction are highly interconnected, leading to severe pathogenic conditions. *NRF2*, being a master regulator of stress responses, has been shown to be activated in diverse tumor types where it is implicated in tumor formation, progression, and metastasis [120,121]. NRF2 forms heterodimers with MAF proteins and regulates the expression of multitudes of genes that are under the control of antioxidant response elements (ARE) [122]. The functions of *NRF2* in cancer include both protective and promoting roles. Interestingly, transient activation of NRF2 works against cancer development, while its permanent activation is committed to carcinogenesis [123], metastasis, and resistance to therapies [117,124].

In neuroblastoma, *NRF2* has been described to promote cell proliferation and resistance to retinoic acid (RA) cytotoxicity [125], as well as to a variety of other therapeutic agents. In respect to *MYCN*, a study showed that the aggressive neuroblastoma cell line HTLA-230 bearing *MYCN* amplification was slightly sensitive to bortezomib (BTZ), a selective inhibitor of the proteasome, due to *NRF2* activation and subsequent upregulation of the antioxidant heme oxygenase-1 (HO-1). After treatment, these cells downregulated p53 and upregulated p21, favoring cell survival. However, the SH-SY-5Y cells without *MYCN* amplification exhibited a higher sensitivity to BTZ because they were unable to upregulate HO-1, indicating the importance of *NRF2* in targeting neuroblastoma chemoresistance and driving cell adaption to oxidative stress [126].

The *STAT5B* gene, also known as Signal Transducer and Activator of Transcription 5B, resides at Chromosome 17q21.2 in a genomic region of 77.2 kb. *STAT5B* encodes for a TF that optimally binds to tandemly arranged interferon gamma-activated sequence (GAS) motifs [127]. Prior to activation, STAT5B is located in the cytoplasm. Upon activation, it becomes phosphorylated, translocates into the nucleus, and regulates the transcriptional activation of its target genes [128]. *STAT5B* is executive in hematopoiesis and in the differentiation of pluripotent stem cells (PSCs) in adult bone marrow. *STAT5* plays an oncogenic role through the activation of c-Myc, cyclin D1, and Bcl-xl, inducing cellular transformation and progression of the cell cycle [129].

Its aberrant activation has been associated with myeloid malignancies such as chronic and acute myelogenous leukemia (CML and AML) [130]. *STAT5*’s contribution to metabolic rewiring in cancer has been well-studied. It has been linked to oxidative metabolism in pre-B cells [131] as well as to a metabolic shift towards aerobic glycolysis in tumor cells [132]. Additionally, there is evidence of *STAT5*‘s direct activation of the glutamine synthetase (GS) enzyme upon radiotherapy. The GS promoter region harbors STAT5 response elements, and GS elevated expression in radioresistant cancer cells is suggested to enhance their DNA repair capacity and their survival advantage [133]. Moreover, an interaction of STAT5B with the δ-opioid receptors has been shown to increase neuronal survival, as well as neurite outgrowth and differentiation [134,135]. In neuroblastoma cell lines, *STAT5B* has been reported as a target of 5-aza-2′-deoxycytidine treatment to induce cell apoptosis and inhibit cell proliferation by changing the expression level of genes involved in the JAK/STAT pathway [136], indicating its important role.

## 5. Conclusions

Taken altogether, it is evident that *MYCN* amplification exhibits a crucial regulatory role in neuroblastoma cells, and our findings provide additional mechanistic insights through the regulation of pivotal transcription factors such as *BCL6*, *KLF4*, *DLX1*, *NRF2*, and *STAT5B*, which confer altered gene expression programs. It is known that inducible gene expression requires the synergistic operation of TFs and their counterparts that function together with respect to signal integration and epigenomic dependencies, e.g., accessibility of histone-DNA structures and nucleosome packaging, modifications on histones and DNA, coactivators and chromatin modifier/remodeler integration in chromosomal landscapes, etc. [16,137]. In many cases, a TF is sufficient to reprogram a cellular state or even to transform entire tissues or organs, e.g., HOX TFs [138]. Such transformations are gained through the establishment of newly developed gene expression programs that efficiently convert the phenotypic characteristics of origin. In neuroblastoma, this phenotypic conversion primarily relies, among others, on the overexpression of MYCN oncoprotein, which accounts for the altered transcriptional regulation of dozens of genes. Elegant studies have shown that, even when “chopped out” from the endogenous chromatin microenvironment, *MYCN* can be assembled in ecDNA amplicons, which therefore episomally drive the overexpression of *MYCN* [39]. Here, we show that occasionally in *MYCN*-amplified oncogenotypes, some of the upregulated *MYCN* target-genes encode for additional TFs, and many of those share the capacity to implement metabolism-related functions. Given the capability of TFs to establish regulatory networks [18], to fine-tune their own expression by assembling auto-regulatory loops [16], and to execute distinct modalities depending, among others, on the epigenetic landscape of incorporation, it becomes evident that neuroblastoma oncophenotype evolution might be mechanistically controlled in multiple layers. Moreover, it is well respected that an advanced approach to developing therapeutic applications targets the fundamental components that regulate the dysregulated gene expression programs on which the oncogenic phenotype relies [16]. A class of such functional candidate molecules is the TFs, which in many cases function as the master regulators of tumorigenesis or downstream targets of the oncogenic drivers. This provides tremendous capabilities for designing therapeutic approaches based on synthetic lethality [139]. Such vulnerabilities cannot be identified or precisely predicted by plain DNA sequence examinations but require an in-depth understanding of how cancer cells establish their vital gene expression programs. Hence, we envision that monitoring of the TFs’ mechanistic dependencies and functional interrelationships is essential in order that such perplexing biological phenomena of human pathogenesis will become adequately disentangled.

## Data Availability

RNA-sequencing FASTQ files of human neuroblastoma cell lines were downloaded from the Gene Expression Omnibus (GEO; https://www.ncbi.nlm.nih.gov/geo/, accessed on 21 June 2023) database (GEO accession number GSE90683), and information about samples can be found in Boeva, et al., 2017 [47]. RNA-sequencing FASTQ files of human neuroblastoma patient-derived samples were downloaded from the Gene Expression Omnibus (GEO; https://www.ncbi.nlm.nih.gov/geo/, accessed on 21 June 2023) database (GEO accession number GSE94035), and information about samples can be found in Rifatbegovic et al., 2018 [52]. RNA-seq data for TARGET-NBL datasets with related clinical information were retrieved from the National Cancer Institute GDC Data Portal. ChIP-sequencing FASTQ files for transcription factor *MYCN* in human neuroblastoma cell lines were obtained from the Gene Expression Omnibus (GEO; https://www.ncbi.nlm.nih.gov/geo/, accessed on 21 June 2023) database (GEO accession numbers GSE138315 and GSE199086). Further information regarding the samples can be found in Upton et al., 2020 [29] and Wang et al., 2023 [26]. The ChIP-sequencing FASTQ file for transcription factor *ASCL1* in human neuroblastoma cell line with *MYCN* amplification was sourced from the Gene Expression Omnibus (GEO; https://www.ncbi.nlm.nih.gov/geo/, accessed on 21 June 2023) database (GEO accession number GSE120074). Detailed sample information can be found in Wang et al., 2019 [55].

## Supplementary Materials

The following supporting information can be downloaded at: https://www.mdpi.com/article/10.3390/cancers15194803/s1, Figure S1: Differential gene expression analyses in neuroblastoma: (A) The dotplot illustrates the most statistically and biologically significant processes associated with the total spectrum of 286 uDEGs, as defined by GOs. The metabolism-related ontologies are highlighted within boxes. (B) The heatmap depicts the transcriptional profile of TF-encoding genes side by side in non-*MYCN*-amplified and *MYCN*-amplified neuroblastoma cell lines. Figure S2: ASCL1-binding profile and correlation with neuroblastoma metabolism. (A) Intersection of ASCL1- and MYCN-ChIP-seq peaks and GOs on their common spectrum. Metabolism-related processes are highlighted. (B) The distribution of ASCL1 proximal to metabolism-related TFs in Kelly neuroblastoma cells. Several binding events are captured proximal to the TSSs of *GLI2*, *HLF*, *KLF4*, *NRF2*, *NR1D1*, *NR2F2*, *TCFL5*, *ZFP1*, and *MYCN*. Figure S3: Transcriptional profiling of *MYCN*-amplified and non-amplified neuroblastoma patient-derived samples. (A) Gene expression investigation on *MYCN*-amplified and non-*MYCN*-amplified stage 4/M metastatic neuroblastoma specimens. (i) Selected examples of the most statistically and biologically significant biological processes, as determined by gene ontology analyses (GOs) applied to the 1396 uDEGs, are highlighted. Among these, several ontologies and functions connected to metabolism are depicted. (ii) RNA-seq heatmap illustrating the transcriptional upregulation of *MYCN*, *BCL6*, *KLF4*, *SIX4*, *NR2F2*, *GLI2*, *GLI3*, and *WT1*. The heatmap was generated using log_2_(CPM) normalized counts. (B) Gene expression investigation on *MYCN*-amplified and non-*MYCN*-amplified primary, undifferentiated or poorly differentiated, high-risk, stage 4, neuroblastoma specimens. (i) Selected examples of the most statistically and biologically significant biological processes, as determined by gene ontology analyses (GOs) applied to the 3424 uDEGs, are highlighted. Among these, several ontologies and functions connected to metabolism are depicted. (ii) RNA-seq heatmap illustrating the transcriptional upregulation of *FOXF1*, *OSR2*, *WT1*, *MYCN*, *NR2F2*, *SIX4*, *GLI2*, *NR1D1*, *HLF*, and *CREBZF*. The heatmap was generated using log_2_(CPM) normalized counts. Table S1: Differential gene expression analysis applied in *MYCN*-amplified compared to non-*MYCN*-amplified neuroblastoma cell lines and TFs list. Table S2: MYCN-inducible binding signals (peaks) enriched in *MYCN*-amplified and *MYCN*-overexpressing cell lines retrieved from our MYCN-ChIP-seq meta-analyses. Table S3: Differential gene expression analysis applied in *MYCN*-amplified compared to non-*MYCN*-amplified neuroblastoma patient-derived samples and TFs list.
