# Supplementary material for: MYCN Amplifications and Metabolic Rewiring in Neuroblastoma
Source: Cancers (Basel). 2023 Sep 29;15(19):4803. doi: 10.3390/cancers15194803 (PMC10571721; doi:10.3390/cancers15194803)
Supplement: Supplementary file 1 [file cancers-15-04803-s001.zip › Supplementary Figures S1-S3.pdf]

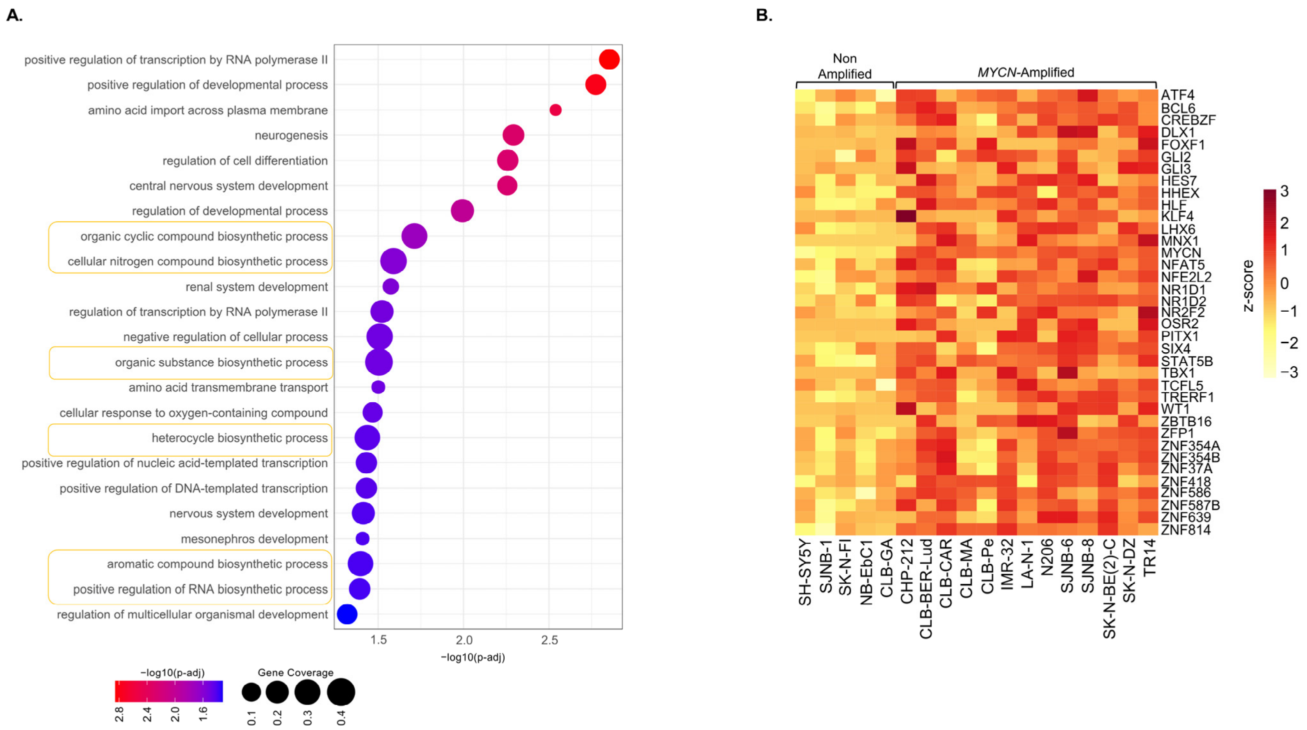


**Figure S1.** Differential gene expression analyses in neuroblastoma: (A) The dotplot illustrates the most statistically and biologically significant processes associated with the total spectrum of 286 uDEGs, as defined by GOs. The metabolism-related ontologies are highlighted within boxes. (B) The heatmap depicts the transcriptional profile of TF-encoding genes side by side in non-*MYCN*-amplified and *MYCN*-amplified neuroblastoma cell lines.

A.

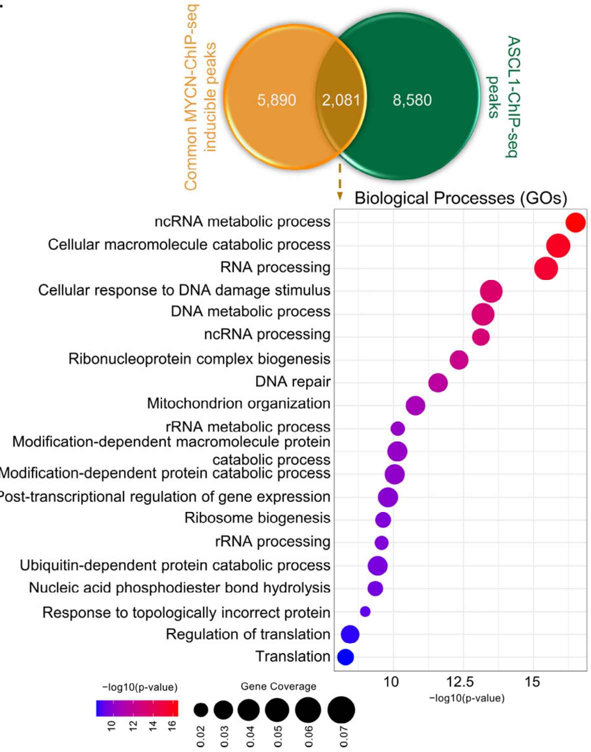


B.

**ASCL1-ChIP-seq peaks in Kelly neuroblastoma cells**

| Genomic coordinates | Neighboring metabolic-related TF gene | Distance from TSS in bp (GREAT) |
|---|---|---|
| chr2:121,538,192-121,538,293 | GLI2 | −11,742 |
| chr2:121,546,237-121,546,388 | | −3,672 |
| chr2:121,567,424-121,567,641 | | +17,548 |
| chr2:121,577,845-121,578,069 | | +27,972 |
| chr2:121,660,179-121,660,280 | | +110,245 |
| chr2:121,696,104-121,696,220 | | +146,177 |
| chr17:53,342,088-53,342,236 | HLF | −211 |
| chr9:110,252,211-110,252,356 | KLF4 | −357 |
| chr2:16,079,857-16,080,083 | MYCN | −716 |
| chr2:16,080,162-16,080,561 | | −324 |
| chr2:16,081,702-16,081,876 | | +1,103 |
| chr2:16,083,551-16,083,882 | | +3,031 |
| chr2:16,087,633-16,087,836 | | +7,049 |
| chr2:178,129,554-178,129,681 | NFE2L2 (NRF2) | +241 |
| chr17:38,256,881-38,257,024 | NR1D1 | +25 |
| chr15:96,873,776-96,873,877 | NR2F2 | −119 |
| chr15:96,884,086-96,884,245 | | +10,220 |
| chr20:61,493,351-61,493,471 | TCFL5 | −296 |
| chr16:75,182,843-75,182,947 | ZFP1 | +415 |

**Figure S2.** ASCL1-binding profile and correlation with neuroblastoma metabolism. (A) Intersection of ASCL1- and MYCN-ChIP-seq peaks and GOs on their common spectrum. Metabolism-related processes are highlighted. (B) The distribution of ASCL1 proximal to metabolism-related TFs in Kelly neuroblastoma cells. Several binding events are captured proximal to the TSSs of *GLI2*, *HLF*, *KLF4*, *NRF2*, *NR1D1*, *NR2F2*, *TCFL5*, *ZFP1*, and *MYCN*.

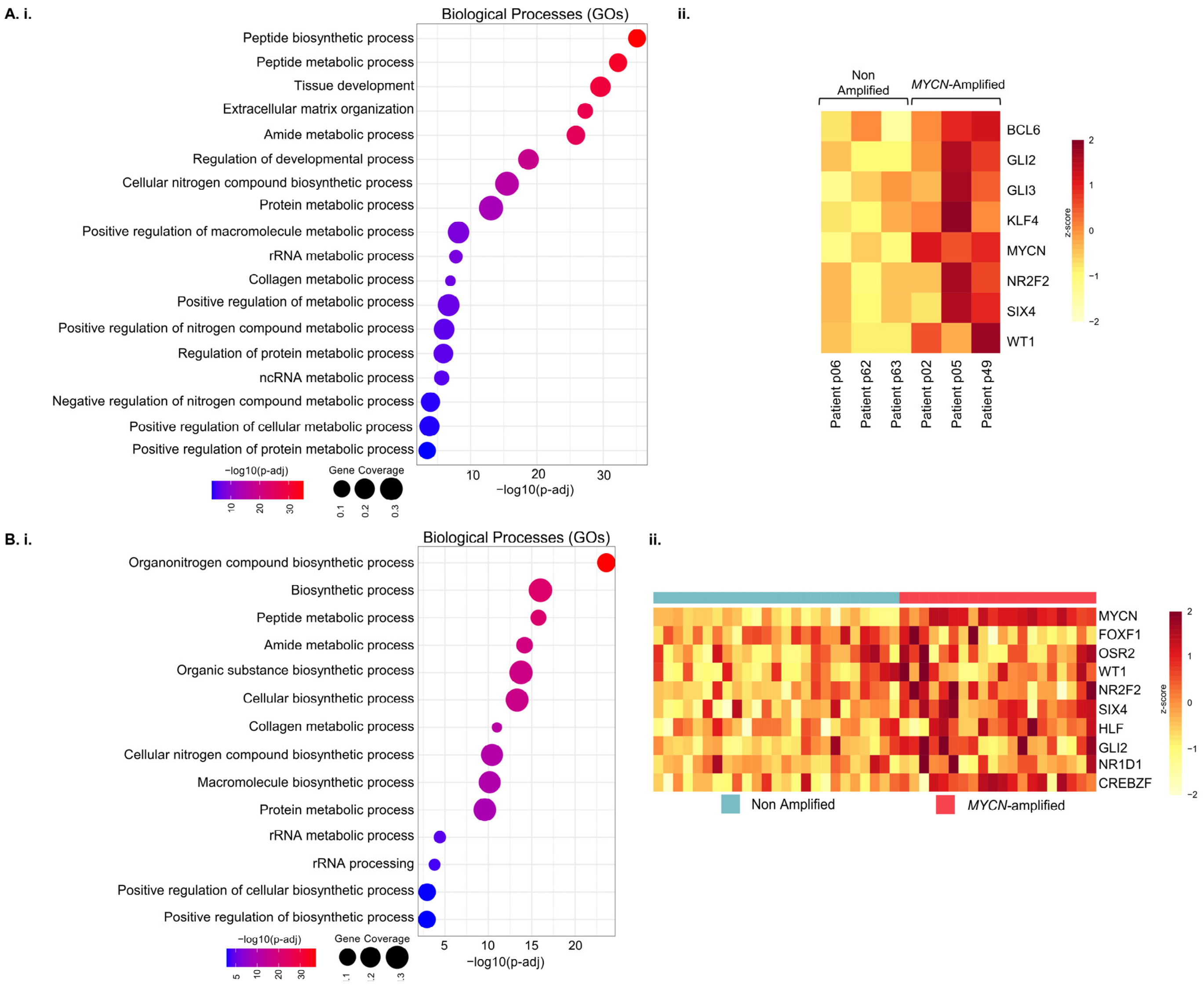


**Figure S3.** Transcriptional profiling of *MYCN*-amplified and non-amplified neuroblastoma patient-derived samples. (A) Gene expression investigation on *MYCN*-amplified and non-*MYCN*-amplified stage 4/M metastatic neuroblastoma specimens. (i) Selected examples of the most statistically and biologically significant biological processes, as determined by gene ontology analyses (GOs) applied to the 1396 uDEGs, are highlighted. Among these, several ontologies and functions connected to metabolism are depicted. (ii) RNA-seq heatmap illustrating the transcriptional upregulation of *MYCN*, *BCL6*, *KLF4*, *SIX4*, *NR2F2*, *GLI2*, *GLI3*, and *WT1*. The heatmap was generated using $log_2$(CPM) normalized counts. (B) Gene expression investigation on *MYCN*-amplified and non-*MYCN*-amplified primary, undifferentiated or poorly differentiated, high-risk, stage 4, neuroblastoma specimens. (i) Selected examples of the most statistically and biologically significant biological processes, as determined by gene ontology analyses (GOs) applied to the 3424 uDEGs, are highlighted. Among these, several ontologies and functions connected to metabolism are depicted. (ii) RNA-seq heatmap illustrating the transcriptional upregulation of *FOXF1*, *OSR2*, *WT1*, *MYCN*, *NR2F2*, *SIX4*, *GLI2*, *NR1D1*, *HLF*, and *CREBZF*. The heatmap was generated using $log_2$(CPM) normalized counts.
